# Association between plasma fatty acids and inflammatory markers in patients with and without insulin resistance and in secondary prevention of cardiovascular disease, a cross-sectional study

**DOI:** 10.1186/s12937-018-0342-1

**Published:** 2018-02-21

**Authors:** Ângela Cristine Bersch-Ferreira, Geni Rodrigues Sampaio, Marcella Omena Gehringer, Elizabeth Aparecida Ferraz da Silva Torres, Maria Beatriz Ross-Fernandes, Jacqueline Tereza da Silva, Camila Ragne Torreglosa, Cristiane Kovacs, Renata Alves, Carlos Daniel Magnoni, Bernardete Weber, Marcelo Macedo Rogero

**Affiliations:** 1 Research Institute, Hospital do Coração (IP-HCor), São Paulo, SP Brazil; 2 Department of Nutrition, School of Public Health, University of São Paulo, São Paulo, Brazil; 3 Department of Clinical Nutrition at Instituto Dante Pazzanese de Cardiologia, São Paulo, Brazil; 4 University of São Paulo (USP), Av Dr Arnaldo, 715, São Paulo, SP 01246-904 Brazil

**Keywords:** Polyunsaturated fatty acid, Cardiovascular diseases, Secondary prevention, Inflammation, Insulin resistance

## Abstract

**Background:**

Proinflammatory biomarkers levels are increased among patients with cardiovascular disease, and it is known that both the presence of insulin resistance and diet may influence those levels. However, these associations are not well studied among patients with established cardiovascular disease. Our objective is to compare inflammatory biomarker levels among cardiovascular disease secondary prevention patients with and without insulin resistance, and to evaluate if there is any association between plasma fatty acid levels and inflammatory biomarker levels among them.

**Methods:**

In this cross-sectional sub-study from the BALANCE Program Trial, we collected data from 359 patients with established cardiovascular disease. Plasma fatty acids and inflammatory biomarkers (interleukin (IL)-1β, IL-6, IL-8, IL-10, IL-12, high sensitive C-reactive protein (hs-CRP), adiponectin, and tumor necrosis factor (TNF)-alpha) were measured. Biomarkers and plasma fatty acid levels of subjects across insulin resistant and not insulin resistant groups were compared, and general linear models were used to examine the association between plasma fatty acids and inflammatory biomarkers.

**Results:**

Subjects with insulin resistance had a higher concentration of hs-CRP (*p* = 0.002) and IL-6 (*p* = 0.002) than subjects without insulin resistance. Among subjects without insulin resistance there was a positive association between stearic fatty acid and IL-6 (*p* = 0.032), and a negative association between alpha-linolenic fatty acid and pro-inflammatory biomarkers (*p* < 0.05). Among those with insulin resistance there was a positive association between monounsaturated fatty acids and arachidonic fatty acid and adiponectin (*p* < 0.05), and a negative association between monounsaturated and polyunsaturated fatty acids and pro-inflammatory biomarkers (*p* < 0.05), as well as a negative association between polyunsaturated fatty acids and adiponectin (*p* < 0.05). Our study has not found any association between hs-CRP and plasma fatty acids.

**Conclusions:**

Subjects in secondary prevention for cardiovascular disease with insulin resistance have a higher concentration of hs-CRP and IL-6 than individuals without insulin resistance, and these inflammatory biomarkers are positively associated with saturated fatty acids and negatively associated with unsaturated fatty acids.

**Electronic supplementary material:**

The online version of this article (10.1186/s12937-018-0342-1) contains supplementary material, which is available to authorized users.

## Background

Atherosclerotic cardiovascular disease (CVD) such as acute myocardial infarction and stroke remains the leading cause of death worldwide [1]. Therefore, the better understanding of its etiology and of strategies to prevent and to treat CVD is highly needed. It should be noted that inflammatory biomarkers have a positive association with insulin resistance, an important determinant of CVD [2–5]. Also, epidemiological and clinical studies have shown an association between inflammatory biomarkers, such as C-reactive protein (CRP) [6], tumor necrosis factor (TNF)-alpha [7] and interleukin (IL)-6 [8], and risk of cardiovascular events among primary and secondary prevention patients.

Dietary components, especially fatty acids, affect the expression and release of inflammatory biomarkers. Polyunsaturated fatty acids (PUFAs), such as eicosapentaenoic acid (EPA) and docosahexaenoic acid (DHA), have a cardioprotective effect by reducing proinflammatory gene expression (TNF-alpha and IL-1ß [9, 10]), and by increasing gene expression of adiponectin [11, 12], which is protective against vascular dysfunction and insulin resistance [13, 14]. Indeed, clinical studies have shown that diets may have effect on inflammatory biomarkers levels and further effect on insulin sensibility [15, 16]. However, it has been poorly studied among secondary prevention patients, this population has an important characteristic which is they are at high cardiovascular risk and probably well treated (under many drug therapy) [17].

Given the association between inflammation and insulin resistance, and the role of fatty acids on modulating the inflammatory biomarkers levels on primary prevention patients, we aimed to understand if this association might be similar among CVD secondary prevention patients with insulin resistance. Such an association would potentially provide insights into the role of inflammatory biomarkers in different metabolic status of cardiovascular patients. Thus, this cross-sectional study has two objectives: (i) to compare inflammatory biomarker levels among CVD secondary prevention patients with and without insulin resistance; (ii) to evaluate if plasma fatty acid levels are associated with inflammatory biomarker levels among CVD secondary prevention patients with and without insulin resistance.

## Methods

The volunteers are part of the Brazilian Cardioprotective Nutritional Program Trial (BALANCE Program Trial, which is funded by *Hospital do Coração (HCor)* as part of the “*Hospitais de Excelência a Serviço do SUS (PROADI-SUS)*” Program, in partnership with the Brazilian Ministry of Health. All eligibility criteria are reported in the study protocol [18]. Briefly, the BALANCE PROGRAM TRIAL included 2468 patients aged 45 years or older, who had experienced one or more indicators of established CVD in the preceding 10 years.

The initial population considered for this cross-sectional sub-study consisted of 400 volunteers, selected from the BALANCE PROGRAM TRIAL site at the Department of Clinical Nutrition in the *Instituto Dante Pazzanese de Cardiologia*, São Paulo, Brazil. Of these, 41 subjects were excluded due to lack of blood analysis, though the analysis was performed in 359 participants. STROBE-Nut checklist is provided as Additional file 1.

### Ethics

All volunteers were required to read and sign a consent form before their final selection as subjects. The study protocol was approved by the Local Ethics Committee (*Instituto Dante Pazzanese de Cardiologia*, São Paulo, Brazil) and it is in accordance with the Helsinki Declaration principles [19].

### Data collection

#### Sociodemographic, clinical and behavioral characteristics

Trained interviewers administered a structured questionnaire comprising questions on sociodemographic and clinical characteristics. Subjects were classified according to smoking status as current smokers, non-smokers, and former smokers, and activity levels as sedentary and active. Body-mass index (BMI) was calculated as weight (kg)/height (m^2^). Blood pressure was obtained by a trained nurse, following the recommendations of the American Heart Association [20], and medication data were obtained from medical prescriptions. All data were recorded in an electronic case report form.

#### Fatty acids assessment

Total plasma fatty acids (FA) were extracted using a mixture of methanol:chloroform chromatographic solution (2:1, *v*/v), and FA were converted to FA methyl esters using a modified sodium methoxide method. Then, the FA profile was measured using flame-ionization gas chromatography on a device (SHIMADZU, CG-2010, Kyoto, Japan). Samples (2 μL) were injected via an autosampler into a fused-silica capillary column (DB-FFAP capillary column [15 m × 0.100 mm × 0.10 μm] J&W Scientific from Agilent Technologies, Folsom, CA, USA) in a gas chromatography system fitted with a flame ionization detector and eluted with hydrogen at 3.0 mL/min., with a split ratio of 1:150. The injector and detector were heated to 250 °C and 260 °C, respectively. The column was temperature programmed from 100 °C (hold 0.5 min) to 195 °C at 25 °C/min, then to 205 °C (hold 3 min) at 3.0 °C/min. Identification of the fatty acids was achieved by comparing their retention times with pure standards (FAME 37, code 47885, Sigma Chemical Co). Individual peaks were quantified as the area under the peak and results expressed as percentages of the total area of all FA peaks: miristic, palmitic, palmitoleic, stearic, oleic, linoleic, α-linolenic, eicosatrienoic, arachidonic, eicosapentaenoic, docosapentaenoic and docosahexaenoic.

#### Laboratory measurement

All subjects were fasted for at least 12 h (maximum 14 h) before phlebotomy. Glycaemia, triglycerides, total cholesterol and high-density cholesterol levels were measured by an enzymatic colorimetric dry chemistry method (Johnsons & Johnsons, Raritan, USA, VITROS 5600). Low-density cholesterol was determined by Friedewald’s formula. Insulin levels were measured by chemiluminescence immunoassay (Linco Research Incorporated, St. Charles, USA, Lincoplex).

High-sensitivity C-reactive protein (hs-CRP) levels were determined through particle-enhanced immunonephelometry. Plasma IL-1β, IL-6, IL-8, IL-10, IL-12, TNF-α and adiponectin levels were measured using a Multiplex immunoassay (Milliplex, Merck Millipore, MA, USA) according to manufacturer’s instructions.

Insulin resistance was estimated by homeostasis model assessment (HOMA), which was calculated as [glycemia (mMol) x insulin (uU/mL) ÷ 22,5]. The insulin resistance was diagnosed if any of the following conditions were met: BMI > 28.9 kg/m^2^, homeostasis model assessment of insulin resistance (HOMA-IR) > 4.65, or BMI > 27.5 kg/m^2^ and HOMA-IR > 3.60 [21].

### Statistical analysis

Categorical variables were presented as frequencies and were compared with chi-square test. Continuous variables were presented as mean (standard deviation (SD)). General characteristics, biomarker levels and fatty acid plasma concentration of subjects across groups were compared using t-test and Mann-Whitney where appropriate.

After log-transformation of hs-CRP, IL-6, IL-8, IL-10, IL-12, IL-1β, adiponectin and TNF-a, general linear models were used to examine the association between plasma fatty acids and plasma inflammatory markers concentration, stratified by presence or absence of insulin resistance. Gaussian Family general linear model was applied for all analyses, except for the adiponectin model, in which we have used an inverse-gaussian family, general linear model. The model was adjusted for sex, calcium channel blockers, insulin, age, BMI, triglycerides, blood pressure and HOMA-IR. The significance level was set at 5% for two-tailed hypotheses tests for multiple comparisons. Because of this, all analyses should be interpreted as exploratory. All statistical analysis was performed in the R software environment (V.3.1.0, R Core Team, 2014).

## Results

In the present study, 359 patients in secondary prevention of CVD were evaluated (Table 1). Of these, 51% (*n* = 183) had insulin resistance and 49% did not (*n* = 176). Comparing the demographic characteristics between groups (insulin resistance group [IRG] and not insulin resistance group [NIRG]), it was observed that a higher proportion of the NIRG were men (*p* = 0.005). Regarding baseline atherosclerotic cardiovascular diseases, the groups had similar proportions of individuals who were infarcted or who had previously undergone angioplasty. In terms of medication, the NIRG presented a lower proportion of subjects taking the antihypertensive calcium channel antagonist (*p* = 0.006).

**Table 1:** General characteristics of patients across insulin resistance presence

	NIRG (*n* = 176)		IRG (*n* = 183)		
	n	%	n	%	*p*
Sex					
Males	156	88,60%	105	57,40%	0.005
Physical activity					
Sedentary	105	59,70%	117	64,60%	0.595
Active	71	40,30%	66	36,50%	
Clinical characteristics					
Previous myocardial revascularization	142	81%	142	78%	0.472
Hypertension	166	94%	179	98%	0.09
Type 2 diabetes mellitus	81	46%	98	54%	0.154
Dyslipidemia	155	88%	161	88%	0.979
CVD family history	131	74%	127	69%	0.299
Smoking status					
Current smoker	11	6%	11	6%	0.864
Former smoker	97	55%	106	58%	
Nonsmoker	68	39%	66	36%	
Drugs in use					
Acetylsalicylic acid	165	94%	171	93%	0.905
Clopidogrel	11	6%	20	11%	0.114
Beta-blocker	152	86%	155	85%	0.654
Thiazide diuretics	38	22%	56	31%	0.06
ACE inhibitors	86	49%	88	48%	0.883
Angiotensin receptor blockers	47	27%	64	35%	0.09
Calcium channel blockers	38	22%	63	34%	0.006
Statin	166	94%	164	90%	0.102
Metformin	71	40%	82	45%	0.392
Insulin	23	13%	38	21%	0.05

*IRG* group of patients with insulin resistance, *NIRG* group of patients non-insulin resistant, *CVD* cardiovascular disease, *ACE inhibitors* angiotensin-converting-enzyme inhibitor

NIRG subjects were older than IRG (*p* < 0.001) (Table 2). Regarding metabolic clinical characteristics, values for body weight, waist circumference, and BMI were significantly (*p* < 0.001) lower in the NIRG than the IRG. In addition, NIRG individuals had lower plasma concentration of triacylglycerol and insulin than the IRG.

**Table 2:** Mean (SD) of anthropometric and clinical data among IRG and NIRG

	NIRG		IRG		*p*
	mean	sd	mean	sd	
Age, y	66.22	8.93	62.39	9.13	< 0.001
Waist circumference, cm	92.87	7.96	108.58	9.90	< 0.001
Weight, kg	67.91	9.57	87.18	13.7	< 0.001
BMI, kg/m2	25.60	2.30	33.29	3.94	< 0.001
Cholesterol total, mg/dL	160.39	35.30	161.74	36.61	0.72
LDL cholesterol, mg/dL	84.73	28.65	81.75	27.43	0.32
HDL cholesterol, mg/dL	46.78	12.88	44.75	15.80	0.18
Triacylglycerol, mg/dL	142.99	80.49	183.97	125.61	< 0.001
Glycaemia, mg/dL	107.90	37.85	112.78	37.77	0.22
Insulin, mIU/mL	8.19	4.73	15.12	11.88	< 0.001
HOMA	1.11	0.66	1.89	1.20	< 0.001
Systolic blood pressure, mmHg	129.73	20.23	136.85	22.87	< 0.001
Diastolic blood pressure, mmHg	75.27	11.68	81.28	15.55	0.040
CRP, mg/dL	6.00	12.70	7.90	18.60	0.002
Adiponectin, mg/mL	9601.43	6679.80	8149.63	5061.45	0.124
IL-10, pg/mL	21.97	21.62	23.20	22.51	0.499
IL-12, pg/mL	6.88	33.28	4.98	10.70	0.119
IL-1 b, pg/mL	1.11	4.25	2.28	18.27	0.38
IL-6, pg/mL	7.46	13.10	9.04	13.46	0.002
IL-8, pg/mL	4.82	2.78	4.81	2.61	0.964
TNF-a, pg/mL	7.85	5.23	8.49	5.98	0.395
Myristic fatty acid - C14:0 (%)	0.47	0.33	0.60	0.42	0.007
Palmitic fatty acid- C16:0 (%)	24.47	3.50	25.28	3.55	0.07
Palmitoleic fatty acid - C16:1 (%)	1.59	0.74	1.80	0.66	0.001
Estearic fatty acid - C18:0 (%)	12.11	1.67	11.82	1.78	0.18
Oleic fatty acid - C18:1 (%)	20.31	3.80	21.14	3.47	0.07
Linoleic fatty acid - C18:2 (%)	22.85	3.87	22.25	3.38	0.19
α-linolenic fatty acid- C18:3 (%)	0.75	0.33	0.71	0.26	0.322
Eicosatrienoic fatty acid - C20:3 (%)	2.55	0.61	2.57	0.64	0.78
Araquidonic fatty acid -C20:4 (%)	11.42	2.88	10.59	2.61	0.02
Eicosapentaenoic fatty acid - C20:5 (%)	0.74	0.49	0.70	0.29	0.507
Docosapenatenoic fatty acid - C22:5 (%)	0.97	0.27	0.90	0.23	0.02
Docosahexaenoic fatty acid - C22:6 (%)	2.79	1.09	2.53	0.85	0.06

*IRG* group of patients with insulin resistance, *NIRG* group of patients non-insulin resistant, *BMI* body-mass index, *CRP* C-reactive protein, *HDL* high-density lipoprotein, *HOMA* homeostasis model assessment, *IL* interleukin, *LDL* low-density lipoprotein, *TNF* tumor necrosis factor

Data are expressed as mean (standard deviation). *P* < 0.05 indicate the significant difference between groups

Regarding inflammatory biomarkers, we found that the plasma concentration of hs-CRP and IL-6 was significantly lower among the NIRG (*p* < 0.002) than the IRG. NIRG individuals had a lower plasma concentration of myristic fatty acid (*p* = 0.007), palmitoleic (*p* = 0.001), and a higher plasma concentration of arachidonic fatty acid (*p* = 0.02) and docosapentaenoic fatty acid (*p* = 0.02) than the IRG.

Multivariate-adjusted general linear models were used to examine the effect of different plasma fatty acids on plasma inflammatory biomarkers levels (Tables 3 and 4). Among the NIRG (Table 3), proinflammatory cytokines, such as IL-6 and TNF-alpha presented inverse association with alpha-linolenic fatty acid, a polyunsaturated fatty acid, and a direct association with the stearic fatty acid, a saturated fatty acid. Among the IRG (Table 4), there was no association between proinflammatory cytokines and saturated fatty acids, however inverse associations were observed not only between IL-6 and TNF-alph, but also between Il-1β and IL-8 and unsaturated fatty acids.

**Table 3:** Multivariate-adjusted general linear model estimates for the association between plasma fatty acid and inflammatory markers among CVD secondary prevention patients without insulin resistance

	Log (CRP)		Log (Adiponectin)		Log (IL-6)		Log (IL1-B)		Log (IL-8)		Log (IL-10)		Log (IL-12)		Log (TNF-a)	
	Estimator	P	Estimator	P	Estimator	P	Estimator	p	Estimator	p	Estimator	p	Estimator	p	Estimator	p
Miristic fatty acid - C14:0 (%)	−0.410	0.389	0.0001	0.893	0.290	0.383	0.241	0.697	−0.046	0.822	0.035	0.915	0.984	0.127	0.189	0.324
Palmitic fatty acid- C16:0 (%)	−0.056	0.097	0.0000	0.828	0.040	0.086	0.06	0.897	0.002	0.891	− 0.01	0.515	0.066	0.143	0.096	0.178
Palmitoleic fatty acid - C16:1 (%)	0.027	0.882	0.0002	0.487	0.152	0.214	0.151	0.509	0.08	0.286	0.096	0.422	0.244	0.305	0.098	0.166
Stearic fatty acid - C18:0 (%)	−0.058	0.462	−0.0001	0.348	0.114	0.032	0.05	0.609	0.007	0.827	0.05	0.342	0.064	0.538	−0.015	0.618
Oleic fatty acid - C18:1 (%)	0.039	0.241	0.0001	0.234	−0.012	0.588	0.04	0.333	0.016	0.251	0.023	0.284	0.019	0.667	0.012	0.361
Linoleic fatty acid - C18:2 (%)	−0.002	0.940	− 0.0001	0.150	− 0.031	0.111	− 0.04	0.235	− 0.014	0.259	− 0.025	0.174	− 0.053	0.161	− 0.012	0.237
α-linolenic fatty acid- C18:3 (%)	0.127	0.698	0.0003	0.557	−0.579	0.008	−0.717	0.082	−0.115	0.401	−0.519	0.016	−0.822	0.054	−0.283	0.027
Eicosatrienoic fatty acid - C20:3 (%)	0.228	0.216	−0.0006	0.024	0.168	0.178	0.15	0.526	0.077	0.316	0.012	0.313	0.157	0.522	0.079	0.234
Araquidonic fatty acid -C20:4 (%)	−0.071	0.133	0.0001	0.316	0.032	0.308	−0.004	0.94	−0.016	0.427	0.013	0.668	0.021	0.735	−0.004	0.818
Eicosapentaenoic fatty acid - C20:5 (%)	0.051	0.818	0.0000	0.918	−0.273	0.067	−0.359	0.198	0.099	0.284	−0.265	0.07	−0.309	0.288	−0.145	0.093
Docosapenatenoic fatty acid - C22:5 (%)	0.210	0.614	−0.0001	0.828	−0.482	0.089	−0.32	0.544	−0.068	0.699	−0.125	0.656	−0.863	0.121	−0.075	0.646
Docosahexaenoic fatty acid - C22:6 (%)	−0.077	0.482	0.0001	0.346	−0.067	0.366	−0.024	0.862	−0.02	0.651	−0.029	0.715	−0.023	0.87	−0.036	0.399

*CRP* C-reactive protein, *IL* interleukin

Analysis adjusted for sex, previous stroke, calcium channel blockers, insulin, age, BMI, triglycerides, blood pressure and HOMA

**Table 4:** Multivariate-adjusted general linear model estimates for the association between plasma fatty acid and inflammatory markers among CVD secondary prevention patients with insulin resistance

	Log (CRP)		Log (Adiponectin)		Log (IL-6)		Log (IL1-B)		Log (IL-8)		Log (IL-10)		Log (IL-12)		Log (TNF-a)	
	Estimator	P	Estimator	P	Estimator	P	Estimator	p	Estimator	p	Estimator	p	Estimator	p	Estimator	p
Miristic fatty acid - C14:0 (%)	−0.402	0.126	0	0.23	0.257	0.154	0.566	0.095	−0.001	0.99	0.255	0.182	0.188	0.561	0.086	0.467
Palmitic fatty acid- C16:0 (%)	−0.05	0.104	0	0.079	0.019	0.36	0.037	0.354	−0.003	0.837	−0.021	0.337	−0.037	0.335	−0.006	0.638
Palmitoleic fatty acid - C16:1 (%)	0.085	0.629	0.001	0.005	−0.07	0.567	−0.194	0.397	−0.059	0.484	−0.227	0.076	−0.411	0.057	−0.149	0.059
Stearic fatty acid - C18:0 (%)	−0.025	0.705	0	0.097	0.025	0.591	0.029	0.737	−0.058	0.069	0.028	0.561	0.059	0.469	0.001	0.961
Oleic fatty acid - C18:1 (%)	0.033	0.329	0	0.015	−0.011	0.655	−0.0561	0.039	−0.014	0.408	0.003	0.907	−0.036	0.39	−0.011	0.493
Linoleic fatty acid - C18:2 (%)	0.006	0.837	0	0.391	0.03	0.14	0.042	0.274	0.021	0.141	0.021	0.333	0.041	0.258	0.031	0.021
α-linolenic fatty acid- C18:3 (%)	0.009	0.981	0	0.475	−0.071	0.789	0.193	0.699	0.113	0.541	−0.046	0.871	0.289	0.543	0.278	0.111
Eicosatrienoic fatty acid - C20:3 (%)	−0.143	0.394	0	0.314	−0.076	0.513	0.044	0.839	−0.158	0.047	−0.072	0.559	−0.077	0.712	−0.118	0.12
Araquidonic fatty acid -C20:4 (%)	0.017	0.729	0	0.047	−0.03	0.364	−0.006	0.926	0.002	0.919	0.019	0.57	0.066	0.254	0.012	0.583
Eicosapentaenoic fatty acid - C20:5 (%)	0.051	0.88	−0.001	0.011	−0.551	0.015	−0.39	0.402	−0.191	0.23	−0.303	0.211	−0.434	0.289	−0.299	0.045
Docosapentenoic fatty acid - C22:5 (%)	0.136	0.77	−0.001	0.052	−0.845	0.007	−0.473	0.435	−0.163	0.462	−0.137	0.684	0.021	0.717	−0.193	0.359
Docosahexaenoic fatty acid - C22:6 (%)	0.043	0.729	0	0.303	−0.101	0.231	0.064	0.706	−0.053	0.363	−0.038	0.669	0.047	0.753	−0.036	0.399

*CRP* C-reactive protein, *IL* interleukin

Analysis adjusted for sex, previous stroke, calcium channel blockers, insulin, age, BMI, triglycerides, blood pressure and HOMA

There was no significant association between plasma concentrations of hs-CRP and IL-12, and plasma fatty acids in either NIRG (Table 3) or IRG (Table 4). Among the NIRG (Table 3), eicosatrienoic fatty acid had a negative association with plasma adiponectin concentration (*p* = 0.024). This negative association was not found in the IRG, on the other hand, other four fatty acids showed association with adiponectin among IRG. Plasma adiponectin concentration was negatively associated with the eicosapentaenoic fatty acid (EPA) (*p* = 0.011) (Table 4), while the oleic and palmitoleic monounsaturated fatty acids and the arachidonic fatty acid were positively associated with the adiponectin plasma concentration (*p* = 0.015, *p* = 0.005, *p* = 0.047, respectively).

In the IRG, EPA and docosapentaenoic fatty acids were negatively associated with IL-6 plasma concentration (*p* = 0.015 and *p* = 0.007, respectively). For every one unit increase of plasma EPA level (1%), there was a reduction of 0.55 on the log(IL-6) mean values; in another perspective, the increase of 1% in EPA reduces on average 40% the IL-6 mean, independently of other variables (Table 3). In the NIRG IL-6 was positively associated with stearic fatty acid concentration (*p* = 0.032) and negatively associated with α-linolenic fatty acid (*p* = 0.008). In another perspective, in the NIRG the increase of 1% in stearic fatty acid increase on average 10% the IL-6 and the increase of 1% in alpha-linolenic fatty acid reduces on average 45% the IL-1 β.

There were no associations between fatty acids and IL-1β and IL-8 in the NIRG. However, in the IRG, the IL-1β was negatively associated with oleic fatty acid (*p* = 0.039), the increase of 1% in oleic fatty acid reduces on average 5% the IL-1β. Furthermore, in the IRG, the increase of 1% in eicosatrienoic fatty acid reduces on average 15% the IL-8.

A negative association between IL-10 plasma concentration and alpha-linolenic fatty acid (*p* = 0.016) was seen in the NIRG. No association between IL-10 and fatty acid in the IRG was found. The TNF-a plasma concentration was positively associated with linoleic fatty acid (*p* = 0.021) and negatively with EPA (*p* = 0.045) in the IRG, which means that the increase of 1% in EPA reduces in average 25% the TNF-alpha. A negative association between TNF-a and alpha-linolenic fatty acid was observed among the NIRG (*p* = 0.027).

## Discussion

In the present cross-sectional study with subjects in secondary prevention for CVD, it can be seen that those who do not have insulin resistance have significantly lower anthropometric indices, as well as a lower plasma concentration of triacylglycerol, insulin and blood pressure. There is compelling evidence that CVD risk factors are present to a significantly greater degree in the subset of overweight/obese individuals that are also insulin resistant [22]. In the present study, we could also observe that patients on secondary prevention for CVD with insulin resistance have higher inflammatory biomarker concentrations than those patients on secondary prevention without insulin resistance.

This higher pro-inflammatory biomarkers concentration may be due to the excess of adipose tissue, especially visceral tissue, and high intakes of certain saturated fatty acids which can enhance the expression of genes encoding proinflammatory proteins such as TNF-alpha and IL-1β, which, in turn, impair the insulin signaling pathway [23].

Saturated fatty acid high plasma concentration, mostly palmitic fatty avid, has a proinflammatory and atherogenic role since it has an association with the toll-like receptor (TLR)-4 and the consequent activation of transcription factors such as NF-κB and AP-1, which induces the synthesis of pro-inflammatory cytokines such as IL-6 and TNF-α [24]. In addition, saturated fatty acid induces insulin resistance due to its antagonistic action on the PPAR-γ coactivator (PGC1-alpha), which promotes the expression of mitochondrial genes involved in oxidative phosphorylation and insulin-mediated glucose uptake [25, 26]. In the present study, in fact, individuals with insulin resistance had a higher plasma concentration of myristic-saturated fatty acid, a fatty acid typically found in dairy products. A systematic review with meta-analysis [27] showed that lauric fatty acid (C12: 0) has the greatest capacity to promote increased plasma concentration of LDL-c, followed by myristic acid (C14: 0) and palmitic acid (C16: 0).

The omega-3 PUFA family, including EPA and DHA, have an anti-inflammatory role and in the present study, a lower plasma concentration of PUFA was observed among IRG. Among the possible mechanisms for the anti-inflammatory effects of PUFA is the activation of peroxisome proliferator-activated receptors (PPAR), which affect the decrease of the NF-kB translocation into the nucleus, thus reducing the expression of pro-inflammatory genes [9, 10, 28]. In the present study, the plasma concentrations of omega-6 PUFAs were positively associated with the plasma concentration of TNF-alpha in the IRG. These fatty acids are considered proinflammatory because they are the main precursors of prostaglandins and leukotrienes with greater biological activity than those from the omega-3 series [29, 30]. However, subjects with high omega-6 intake do not appear to have elevated concentrations of inflammatory biomarkers; indeed, it seems there are not enough data to support the detrimental effect of low omega-6 intake on cardiovascular health [31]. Thus, further studies should be performed among individuals with and without insulin resistance for further clarification of this possible association.

In addition to the increase in saturated fatty acid concentration, IRG also showed an increase in the concentration of hs-CRP and IL-6, which may indicate an increase in cardiovascular risk, given the role that these cytokines play in atherogenesis. IL-6 acts in the chemotaxis and mitogenesis of smooth muscle cells [32], being the main cytokine to stimulate the hepatic synthesis of positive acute phase proteins, such as CRP, which is now known to be an independent, major risk marker of cardiovascular complications [33]. In addition, IL-6 has recently been proposed to play a central role in the link between obesity, inflammation and coronary heart disease [8, 34]. IL-6 production by adipose tissue could directly affect liver metabolism by inducing very low-density lipoprotein (VLDL) secretion and hypertriglyceridemia since the visceral adipose tissue is closely connected to the liver by the venous portal system [35]. Studies have suggested that IL-6 could be involved in insulin resistance and its complications [36, 37]. Considering the results from the comparative analysis between plasma fatty acid concentration and inflammatory biomarkers among the IRG and NIRG groups, we evaluated whether concentrations of plasma fatty acids were associated with an increase or reduction of inflammatory biomarkers in these two groups. We emphasize that significant associations between these variables were most frequently observed among the IRG group, which may demonstrate the importance of the role and impact of nutritional management to reduce cardiovascular risk among these individuals. We noted that, although the IRG group had higher plasma concentration of myristic fatty acid, hs-CRP, and IL-6, there was no association between these variables in either the NIRG or IRG. On the other hand, PUFAs were inversely associated with the plasma concentration of IL-6 in both groups. Furthermore, in the IRG group, plasma stearic saturated fatty acid concentration was positively associated with the plasma concentration of IL-6.

There was no difference in adiponectin concentrations between the IRG and the NIRG. Possibly, this result is associated with the fact that 67.0% of individuals without insulin resistance are already overweight. In addition to the increase in pro-inflammatory protein expression, the visceral adipose tissue increase is related to a reduction in adiponectin expression and synthesis [38]. On the other hand, weight loss and increased PUFA intake are associated with increased adiponectin concentration [39, 40]. The increase in this adipocytokine is associated with an improvement in insulin sensitivity in liver, muscle and adipose tissue [41], as it inhibits the NF-kB signaling pathway [42].

In the present study, a higher plasma concentration of PUFAs was observed in the NIRG group. In the same group, these fatty acids were negatively associated with both inflammatory biomarkers such as IL-6 and TNF-alpha and anti-inflammatory markers such as adiponectin and IL-10. Previously published studies highlight the absence of an association between polyunsaturated fatty acid and adiponectin in patients in secondary prevention for CVD [43, 44]. Why some studies have not found this association is not clear, but it seems to be linked to the fact that these patients are well medicated with drugs that may affect inflammatory biomarker activities [45, 46].

To our knowledge, this is the first study to analyze the difference in characteristics between individuals with and without insulin resistance in secondary prevention for cardiovascular disease. It should be borne in mind that these individuals are at high cardiovascular risk and probably well treated. However, in the present study, we observed that even when medicated, subjects in secondary prevention with insulin resistance presented uncontrolled cardiovascular risk factor. The BALANCE Program nutritional prescription follows the Brazilian guidelines for the treatment of CVD The BALANCE Program diet is designed to meet the nutritional recommendations proposed by the Brazilian Cardiovascular Guidelines, which, in turn, are guided by the nutritional composition of diets such as the Mediterranean diet and the DASH diet. The proposal of is to ensure adequate nutrient intake with a locally appropriate diet, ie, one consisting of foods that are consumed in Brazil, not necessarily the foods consumed as part of the Mediterranean diet. We highly recommend that a subgroup analysis should be realized in the end of BALANCE Trial, regarding the effect of this intervention among those with and without insulin resistance.

However, some limitations deserve consideration. Due to the cross-sectional design, the possibility of residual confounding (such as unknown confounders) cannot be excluded. Even though we have adjusted for confounder by including it in our model, it may still have residual confounding. Only randomization can eliminate unknown confounders. However, once it is well known the effect of fatty acids, especially polyunsaturated fatty acids, on cytokines levels among primary prevention patients for cardiovascular disease [47], we suggest it is not a source of false positive result, it would not bias the estimates toward the null. In addition, we have analyzed 12 different fatty acids, if possible other fatty acids should also be studied. Another limitation would be the lack of dietary intake information in the present study. In the BALANCE Trial, baseline food intake data were obtained by two 24-h dietary recall. However, the patients analyzed in the present cross-sectional study have answered to only one 24-h dietary recall. Since for a better food intake analysis, we must use tools such as a food frequency questionnaire and/or several 24-h reminders, we preferred to exclude this data for this manuscript analysis. Therefore, information such as the ingestion of antioxidants and other bioactive compounds that may have anti-inflammatory activity was not evaluated and therefore could not be included in the analysis. Another important limitation to highlight is that in this study, the significance level was 0.05 without adjustment for multiple comparisons. Because of this, all analyses should be interpreted as exploratory.

## Conclusion

Subjects in secondary prevention for cardiovascular disease with insulin resistance have a higher concentration of hs-CRP and IL-6 than individuals without insulin resistance, and these inflammatory biomarkers are positively associated with saturated fatty acids and negatively associated with unsaturated fatty acids.

## Additional file

Additional file 1: **Table S1.** STROBE-nut: An extension of the STROBE statement for nutritional epidemiology. (DOCX 33 kb)

## Abbreviations

BALANCE Program Trial: Brazilian cardioprotective nutritional program trial
BMI: Body mass index
CI: Confidence interval
CRP: C-reactive protein
CVD: Cardiovascular disease
DHA: Docosahexaenoic acid
EPA: Eicosapentaenoic acid
FA: Fatty acids
HOMA-IR: Homeostasis model assessment of insulin resistance
hs-CRP: High-sensitivity C-reactive protein
IL: Interleukin
IRG: Insulin resistance group
NIRG: Not insulin resistance group
PUFAs: Polyunsaturated fatty acids
TNF: Tumor necrosis factor
